# Cardioprotection Is a Delivery Problem: A First-Medical-Contact Framework for Acute Myocardial Infarction

**DOI:** 10.1021/acsptsci.6c00552

**Published:** 2026-09-17

**Authors:** Vibhudutta Awasthi, Tarun W. Dasari, Kenichi A. Tanaka

**Affiliations:** † Department of Pharmaceutical Sciences, 6186 University of Oklahoma College of Pharmacy, 1110 North Stonewall Avenue, Oklahoma City, Oklahoma 73117, United States; ‡ Department of Internal Medicine, Cardiovascular Diseases Section, University of Oklahoma College of Medicine, 800 Stanton L. Young Blvd., Oklahoma City, Oklahoma 73104, United States; § Department of Anesthesiology, University of Oklahoma Health Campus, 920 Stanton L. Young Blvd., Oklahoma City, Oklahoma 73104, United States

**Keywords:** STEMI, ischemia−reperfusion injury, cardioprotection, nanoparticle drug delivery, mitochondrial permeability transition, first medical contact

## Abstract

Dozens of agents that limit reperfusion injury preclinically have failed clinically. These failures are architectural, not biological: the right biology reaches the wrong place, as the wrong molecule, at the wrong time, judged by the wrong end point. We recast each as a design constraint and specify a staged regimen anchored at first medical contact.

## Graphical abstract

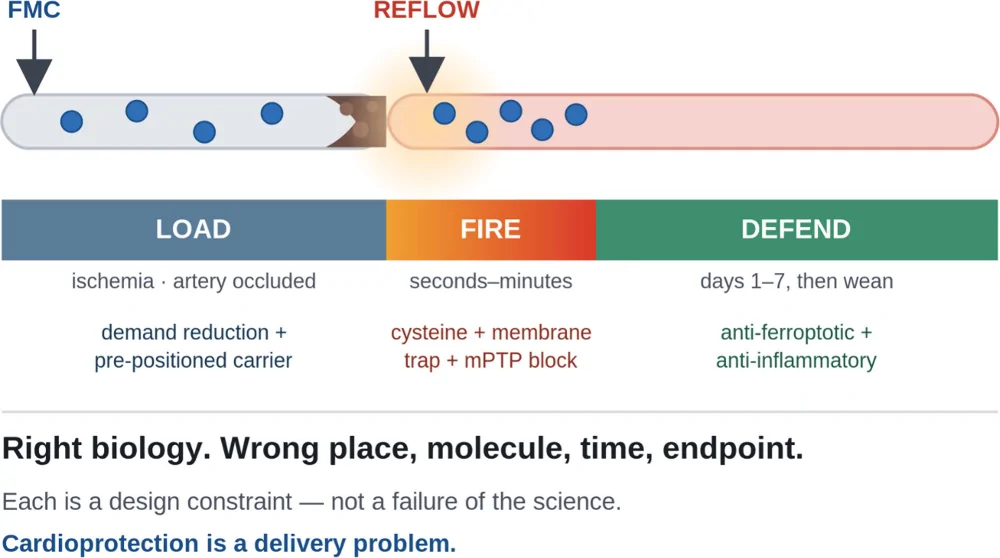

Primary percutaneous coronary intervention (PCI) is the single most important determinant of survival in ST-segment–elevation myocardial infarction (STEMI), yet restoring flow is not purely therapeutic. Few translational failures are as well documented as cardioprotection in revascularized patients. Agents that reliably limit reperfusion injury in animals have done nothing for patients. The usual explanations blame the models. We view the fault as architectural — in where, what, when, and how success is measured — and therefore fixable.

## The Empty Window

Reperfusion sets off a second wave of injury that enlarges the final infarct, and for which no treatment has entered routine practice.^1^ The clinical record catalogs mechanistically sound agents that did not translate to address reperfusion injury (Table 1; Figure 1 ). Cyclosporine, targeting the mitochondrial permeability transition pore (mPTP) through cyclophilin-D, produced an encouraging pilot signal that did not survive the phase-3 CIRCUS trial.^2^ Elamipretide, a cardiolipin-stabilizing agent, failed in EMBRACE-STEMI.^3^ Remote ischemic conditioning, which engages endogenous protection across several pathways, was neutral in CONDI-2/ERIC-PPCI.^4^

**Table 1:** Clinical Cardioprotection Record Set against Preclinical Delivery Proofs-of-Concept^a^

Intervention (trial)	Target/mechanism	Setting and design	Outcome
Cyclosporine — CIRCUS^2^	mPTP/cyclophilin-D	Anterior STEMI, n = 970, phase 3	Neutral: no effect on death, HF, or remodeling at 1 y
Elamipretide — EMBRACE-STEMI^3^	Cardiolipin-targeted mitochondrial antioxidant	Anterior STEMI, phase 2a	Neutral: no reduction in infarct size
Remote conditioning — CONDI-2/ERIC-PPCI^4^	Endogenous multi-pathway conditioning	STEMI, n≈5,400, phase 3	Neutral: no reduction in cardiac death/HF at 1 y
Intravenous metoprolol — METOCARD-CNIC^6^	β1-blockade/demand reduction	Anterior STEMI, Killip ≤II, n = 270	Positive: ↓ infarct size (CMR), ↑ long-term LVEF
Colchicine — COLCOT^10^	Anti-inflammatory (delayed phase)	Post-myocardial infarction, n ≈ 4,745	Positive: ↓ recurrent ischemic events
Pitavastatin-NP (preclinical)^7^	PLGA nanoparticle; PI3K/Akt, anti-inflammatory	Rat ischemia–reperfusion	Positive: ↓ infarct size where free drug failed
CsA-NP + pitavastatin-NP (preclinical)^11^	Dual: mPTP + anti-inflammatory	Murine ischemia–reperfusion	Positive: superior to either single nanoparticle

a Sample sizes are approximate.

The standard failure analysis is by now familiar: low myocardial bioavailability, experiments in young and healthy animals, the comorbidities and polypharmacy that characterize real patients, and trials underpowered for hard end points. Each is true, and none is generative. “Model–patient mismatch” in particular accounts for every failed translation equally well, which is precisely why it discriminates among none of them and prescribes nothing for the next attempt. In our view, a useful diagnosis must convert each obstacle into a specification the next molecule, formulation, or trial can be built against.

We propose that preclinical efficacy is real but is delivered to the wrong place, with the wrong molecule, at the wrong time, and measured with the wrong end point. Each is a design constraint rather than an indictment of the underlying science. Together they identify an actionable and currently empty window: first medical contact (FMC), the interval between paramedic arrival and balloon inflation, during which the artery is still occluded and contemporary care delivers antithrombotics but nothing directed at the reperfusion-injury cascade that begins the instant flow returns.

## Wrong Place: The Delivery Limit Is a Perfusion Limit

Because the culprit artery is occluded, the argument runs, an intravenous drug cannot reach at-risk myocardium during ischemia, so protection is feasible only at reperfusion. This fuses two claims — that drug cannot reach the ischemic core, and that pre-reperfusion intervention therefore cannot help. The first is largely true. The second does not follow.

Consider first whether “no flow” is really no flow. Collateral perfusion governs the transmural depth of necrosis and creates the very gradient that defines the salvageable subepicardial rim;^5^ Thrombolysis in Myocardial Infarction grade 1–2 lesions retain residual antegrade flow; and short-range diffusion reaches inward from perfused borders. These ensure that the jeopardized territory is partly reachable even during occlusion. More decisively, pre-reperfusion intervention demonstrably works: intravenous metoprolol reduced infarct size on cardiovascular magnetic resonance and improved long-term function in anterior STEMI.^6^ This falsifies the futility claim without requiring that any drug reach the core — metoprolol acts on whole-heart workload upstream of the occlusion and on circulating neutrophils, both delivery-independent.

Where a drug must reach tissue, formulation is the lever. Injured myocardium becomes hyperpermeable, so circulating nanoparticles extravasate and are retained in the at-risk zone. Pitavastatin confers no protection given free at reperfusion, yet it becomes cardioprotective when encapsulated in poly­(lactic-*co*-glycolic acid) nanoparticles that accumulate in ischemic-reperfused myocardium.^7^ The “smallness” of a drug is a formulation variable, not a fixed biological barrier. However, enhanced permeability and retention in the infarcted heart is modest and heterogeneous, and the myocardial fraction of an injected nanoparticle dose is small as they are cleared by the mononuclear phagocyte system. Secondly, no intravenously delivered agent reaches tissue with no flow - the truly unperfused subendocardial core, and zones of microvascular obstruction after the epicardial artery is opened. While the unperfused core is addressable only by delivery-independent levers or by pre-positioning a carrier that concentrates at reflow, the obstructed microvasculature is an independent therapeutic target that no formulation can engineer around.

## Wrong Molecule: Redundancy Mandates a Compartment-Matched Combination

Table 1 and Figure 1 establishes the prevailing pattern of searching for a single therapeutic agent, or a single targetable node. This contrasts with the multifactorial nature of post-reperfusion injury.

Reactive oxygen species and calcium each kill through the convergent mPTP and through routes that bypass it — membrane lipid peroxidation and ferroptosis on one side, calpain activation and hypercontracture on the other. A pure pore inhibitor leaves the membrane and calpain routes intact; a pure aqueous-phase scavenger leaves both intramembrane propagation and the calcium, phosphate, and pH triggers of the pore intact. A singly targeted treatment to a node with redundant inputs cannot, even in principle, be sufficient. That cyclosporine failed does not invalidate the mPTP as a target — it is a ″dirty″ agent, with calcineurin off-target activity and solvent confounds — so the case that one node is insufficient rests on the redundancy above. The wider field has reached the same conclusion independently.^8^

Moreover, the antioxidant slot is in two compartments, not one. The initiating species are aqueous: superoxide arising from reverse electron transport is charged, poorly membrane-permeant, and dismutates to freely diffusible hydrogen peroxide. Lipid peroxidation is what follows when that oxidative load meets labile iron at the membrane interface. An antioxidant approach should counter both aqueous radicals and lipid oxidants. While cysteine counters aqueous oxidants and de novo glutathione sustains glutathione peroxidase 4 — the only enzyme that reduces phospholipid hydroperoxides within cellular membranes — neither intercepts the chain-carrying peroxyl radical itself. Once initiated, lipid-radical propagation is intramembrane chemistry, terminated only by a lipophilic radical-trapping agent resident in the bilayer.^9^

A rational regimen therefore needs four arms, each defined by where and when it must act: (i) an aqueous-phase cysteine donor, pre-positioned to raise the threshold for initiation; (ii) a membrane-resident radical-trapping antioxidant, aimed at the ferroptotic bypass a cytosolic thiol cannot reach;^9^ (iii) a specific mPTP/cyclophilin-D modulator, cleaner than cyclosporine, engaged in the first minutes of reflow; and (iv) a delayed-phase anti-inflammatory arm, because salvage that is not defended over the following days is not durable — low-dose colchicine reduced recurrent ischemic cardiovascular events after infarction.^10^

A delivered version of exactly this logic already exists preclinically: a nanoparticle co-formulating a cyclophilin-D inhibitor with an anti-inflammatory statin augmented protection beyond either single-agent nanoparticle.^11^ The redundancy that defeats each monotherapy is thus the affirmative case for a compartment-matched combination. Its components must reach one place but act in several windows — a staging problem that a single programmable carrier can solve.

**Figure 1:**
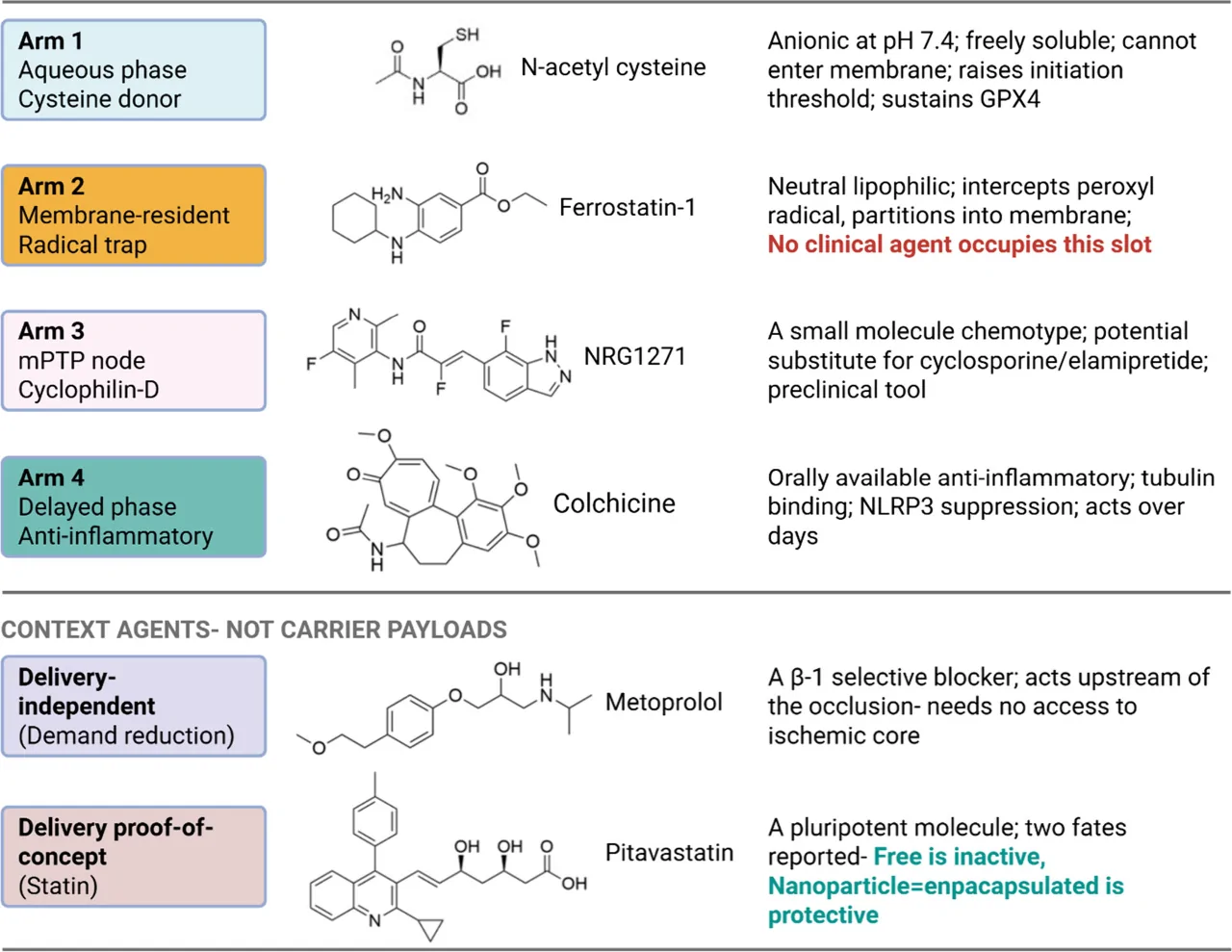
Structures of the molecules discussed in the text, grouped by the compartment their physicochemistry allows them to occupy; the targets and trial outcomes are given in Table 1. One exemplar is shown per arm of the proposed regimen. Arm 2 is occupied by no clinical agent. Pitavastatin illustrates the delivery thesis — the same molecule is inactive free and cardioprotective when nanoparticle-encapsulated. GPX4 = glutathione peroxidase 4.

## Wrong Time: The Staged FMC Regimen

Reperfusion is conventionally treated as the intervention window. We argue it is too narrow for a metabolic reason. Succinate accumulates during ischemia and, the moment oxygen returns, is oxidized in a burst that drives reactive oxygen species production through reverse electron transport at complex I.^12^ In the reperfused myocardial injury, ischemia is the loading phase, and reperfusion is the firing phase (Fig. 2). Because the radical chemistry at reflow is effectively instantaneous, a scavenger cannot be chased into the burst — it must already be resident in the target compartment before flow returns. That is what legitimizes intervening during occlusion even though the lethal oxidative event happens later. As defined in Fig. 2, sorting agents by when they act is what turns a cocktail into a regimen.

**Figure 2:**
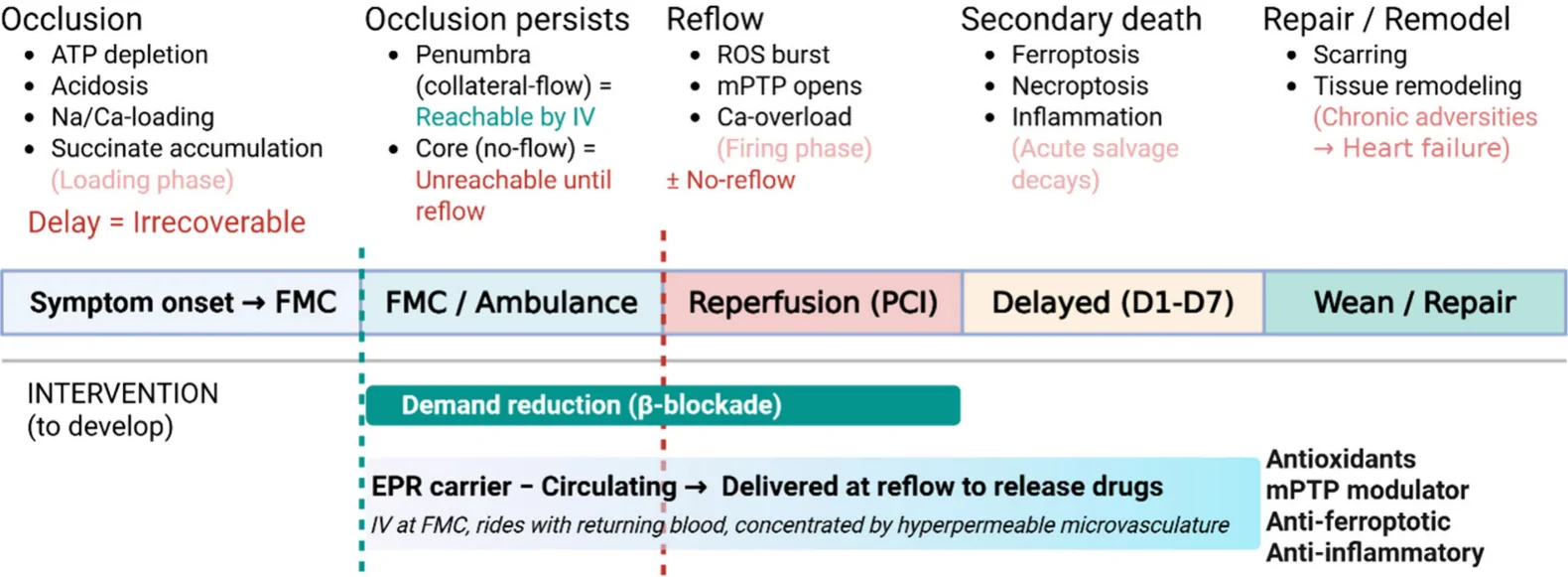
Staged first-medical-contact (FMC) cardioprotection framework mapped to the ischemia → reperfusion → repair clock. Ischemia “loads” the injury (ATP depletion, Na^+^/Ca^2+^ overload, succinate accumulation); reflow “fires” it. Demand reduction (β-blockade) is delivery-independent, lowering the core’s workload. The carrier (blue) is loaded intravenously at FMC, circulates inertly during occlusion, and is concentrated in the injured zone at reflow, releasing reperfusion-instant payloads (aqueous cysteine donor, membrane radical-trapping antioxidant, mPTP/cyclophilin-D modulator) and delayed-phase payloads (anti-ferroptotic and anti-inflammatory), tapered at the inflammation-to-repair transition. The delivery limit is a perfusion limit: the occluded core during ischemia, then no-reflow tissue after it. ATP = adenosine triphosphate; EPR = enhanced permeability and retention; IV = intravenous; mPTP = mitochondrial permeability transition pore; PCI = percutaneous coronary intervention; ROS = reactive oxygen species.

### FMC and Prereperfusion

Two interventions belong here. First is demand reduction to lower whole-heart workload (β-blockade in hemodynamically stable, Killip ≤ II patients).^6^ The second is pre-loading the reperfusion-phase protectants; the carrier with protectants circulates inertly for instant delivery at reperfusion.

### Reperfusion (Seconds to Minutes)

By the time flow returns the payloads are in place, and the carrier is concentrated by the now-hyperpermeable microvasculature. An intracoronary bolus at first flow is the highest-concentration option available and, uniquely, places drug in the territory at the moment no-reflow is being determined.

### Delayed Phase and Wean (Days 1–7)

Ferroptotic injury is enriched at reperfusion but continues to unfold over hours to days. Continued antioxidant and anti-inflammatory coverage defends the salvaged myocardium; it is then withdrawn gradually to allow the inflammation-to-repair transition (reparative healing). Because no marker identifies the inflammation-to-repair transition in a given patient, the taper is prescribed by the carrier’s release kinetics rather than triggered by an event.

One discipline underlies all of this: co-administration is not co-timing. Each agent earns its place only within the window where its mechanism operates. Pharmacokinetic–window mismatch (the right drugs present at the wrong times) is the most likely practical failure of a naïve cocktail, and it is exactly what a staged, programmable carrier is meant to prevent.

## Wrong End Point: The Salvage Trajectory

Preclinical testing relies heavily on a 24 h infarct reading. But death is plural and partly delayed,^9^ so an early benefit that converges on control by 28 days would leave this framework indistinguishable from the monotherapies it was meant to supersede. Therefore, treatment success cannot be a single acute infarct number. The readout should be a salvage trajectory — 24 h, 7 days, and 28–56 days — reported alongside function, in mechanism-enriched infarcts: large, anterior, long ischemic time, poor collaterals, in aged and comorbid animals of both sexes. Microvascular obstruction should be measured but not scored: it confounds any salvage estimate, and a regimen that does not target it should not be judged by it.

The study design must distinguish a genuine combination effect from a better single agent. In other words, every therapeutic agent in the regimen must be shown to earn its place in the presence of the others: withdrawn one at a time from the full combination, rather than tested in isolation against vehicle, with additivity judged against a null stated in advance. Because the carrier arrives as a fixed-dose combination whose payloads cannot be unbundled, the entire burden of component justification falls preclinically.

## So, What Is Missing?

Every pillar of this framework already exists in the literature. The translational-gap diagnosis is established;^1^ the multitarget thesis has a flagship statement;^8^ the delivery axis supplies the load-bearing proof points.^7,11^ The central obstacle is no longer discovery; it is the missing effort to a coordinated assembly. Cardioprotection remains a portfolio of separately owned bets — a mitochondrial peptide, a cyclophilin-D inhibitor, a conditioning protocol, a nanoparticle platform — each at once a legitimate scientific identity and, in practice, a unit of funding, credit, and intellectual property. An integrated program has no single mechanism at its center, and the pharmaceutical industry, the one entity historically able to absorb such a program, has largely withdrawn from a field it perceives as a graveyard after serial late-stage failures.^2,3^

The field has recognized its own fragmentation and responded by building coordinating infrastructure — but of a particular kind. Consortia and position papers integrate methods, end points, and rigor; none is funded to build the multi-component, delivered, staged product that the same consensus implies. An integrator must therefore be an actor whose value lies in the assembly rather than in any single payload — a formulation platform, a consortium, or a public funder, but not a mechanism owner, since no mechanism owner can rationally integrate a competitor’s payload.

## Conclusion

Translation of preclinical cardioprotection has not failed because the biology is wrong. It has failed because the right biology is delivered to the wrong place — the unperfused core rather than the collateral-perfused penumbra; as the wrong molecule — an aqueous-phase thiol (e.g., N-acetyl cysteine) where the chemistry demands a lipophilic terminator of free radical chain (e.g., ferrostatin-1) and a node inhibitor (e.g., NRG1271 or an analogous mPTP-directed chemotype); at the wrong time — a single reperfusion bolus rather than a staged regimen anchored at FMC; and measured with the wrong end point — an acute infarct number that evaporates rather than a durable salvage trajectory. Each is a tractable design constraint. We offer this as a hypothesis-generating design brief: the proposed regimen does not yet exist, and the framework specifies what the next agent should be built to test.

## Abbreviations

Akt: AKR mouse thymoma (also known as protein kinase B)
CMR: Cardiovascular magnetic resonance
CsA: Cyclosporine A
FMC: First medical contact
HF: Heart failure
LVEF: Left-ventricular ejection fraction
mPTP: Mitochondrial permeability transition pore
NP: Nanoparticle
PCI: Percutaneous coronary intervention
PI3K: Phosphoinositide 3-kinase
PLGA: Poly­(lactic-*co*-glycolic acid)
STEMI: ST-segment–elevation myocardial infarction
